# *Francisella tularensis* Confronts the Complement System

**DOI:** 10.3389/fcimb.2017.00523

**Published:** 2017-12-19

**Authors:** Susan R. Brock, Michael J. Parmely

**Affiliations:** Department of Microbiology, Molecular Genetics and Immunology, University of Kansas Medical Center, Kansas City, KS, United States

**Keywords:** cell death, complement, C3, *Francisella tularensis*, macrophage

## Abstract

*Francisella tularensis* has developed a number of effective evasion strategies to counteract host immune defenses, not the least of which is its ability to interact with the complement system to its own advantage. Following exposure of the bacterium to fresh human serum, complement is activated and C3b and iC3b can be found covalently attached to the bacterial surface. However, the lipopolysaccharide and capsule of the *F. tularensis* cell wall prevent complement-mediated lysis and endow the bacterium with serum resistance. Opsonization of *F. tularensis* with C3 greatly increases its uptake by human neutrophils, dendritic cells and macrophages. Uptake occurs by an unusual looping morphology in human macrophages. Complement receptor 3 is thought to play an important role in opsonophagocytosis by human macrophages, and signaling through this receptor can antagonize Toll-like receptor 2-initiated macrophage activation. Complement C3 also determines the survival of infected human macrophages and perhaps other cell types. C3-opsonization of *F. tularensis* subsp. *tularensis* strain SCHU S4 results in greatly increased death of infected human macrophages, which requires more than complement receptor engagement and is independent of the intracellular replication by the pathogen. Given its entry into the cytosol of host cells, *F. tularensis* has the potential for a number of other complement-mediated interactions. Studies on the uptake C3-opsonized adenovirus have suggested the existence of a C3 sensing system that initiates cellular responses to cytosolic C3b present on invading microbes. Here we propose that C3 peptides enter the cytosol of human macrophages following phagosome escape of *F. tularensis* and are recognized as intruding molecular patterns that signal host cell death. With the discovery of new roles for intracellular C3, a better understanding of tularemia pathogenesis is likely to emerge.

*Francisella tularensis* is the bacterial pathogen responsible for the infectious disease tularemia. While tularemia is relatively rare, infection via the respiratory route can be particularly life-threating when not treated with appropriate antibiotics in a timely fashion (Stuart and Pullen, 1945; McCrumb, 1961; Dennis et al., 2001; Feldman et al., 2001). There are two subspecies of *F. tularensis* that account for the majority of infections in immunocompetent human beings. *F. tularensis* subsp. *tularensis* (type A) is considered the more virulent and will be the primary focus of this article. *F. tularensis* subsp. *holarctica* (type B) is also pathogenic in humans, but is less often associated with severe morbidity or mortality. *Francisella novicida* causes a tularemia-like disease in mice, but rarely infects human beings where disease is restricted to the immunocompromised (Kingry and Petersen, 2014). Following exposure to type A and type B *F. tularensis* by the pulmonary route, macrophages are among the first cells infected (Hall et al., 2008; Roberts et al., 2014; Steiner et al., 2017) and serve as an early and continuing replicative niche for the pathogen. Many receptors on the macrophage surface have been implicated in the uptake of *F*. *tularensis* (Clemens et al., 2005; Balagopal et al., 2006; Pierini, 2006; Schulert and Allen, 2006; Barel et al., 2008; Geier and Celli, 2011; Schwartz et al., 2012; Dai et al., 2013), but complement receptors, especially CR3, have consistently been found to be the primary mediators of enhanced uptake of serum-opsonized *F. tularensis* by human macrophages (Clemens et al., 2005; Schwartz et al., 2012; Dai et al., 2013). Once inside the cell, *F. tularensis* escapes the macrophage phagosome at a pace that varies with host species and replicates in the cytosol to high numbers (Golovliov et al., 2003; Clemens et al., 2004; Chong et al., 2008).

Macrophage death is a common outcome following *in vivo* infection with *F. tularensis* and partially explains the appearance of necrotic foci in the livers, lungs, spleens and lymph nodes in several mammalian species (Parmely et al., 2009). The mechanisms and significance of macrophage death depend on the (sub)species of *Francisella* studied (Mariathasan et al., 2005; Henry et al., 2007; Wickstrum et al., 2009). For example, *F. novicida* is a highly proinflammatory pathogen, which induces rapid cell death in mouse macrophages that limits the ability of the bacteria to replicate in the host (Mariathasan et al., 2005; Henry et al., 2007). When describing the lifecycle of type A and type B *F. tularensis*, it is not uncommon to attribute macrophage death to an uncharacterized signal associated with the extensive cytosolic replication of the pathogen. For example, Lai et al. reported on the effects of antibiotic treatment of J774.A1 macrophage-like cells infected with the *F. tularensis* Live Vaccine Strain (LVS) (Lai et al., 2001). Treating cultures with ciprofloxacin within the first 12 h of infection prevented both the replication of the bacteria and host cell death measured 24 h post-infection (PI). If ciprofloxacin treatment was delayed until 15 h PI, host cell death at 24 h PI was similar in magnitude to that of untreated, infected control cells. The authors concluded that intracellular bacterial replication was required for the induction of macrophage death. Recent studies performed in our laboratory with type A *F. tularensis* (Brock and Parmely, 2017) have questioned this interpretation. Intracellular replication of the SCHU S4 strain did not appear to be required for the induction of death in primary human macrophages.

During the course of these studies, we found that complement C3 played an important role in determining the survival of infected macrophages. Accordingly, in this article we review what is known about the interactions between *F. tularensis* and the complement system, discuss recent findings about the functions of intracellular complement, and propose new ways of thinking about the complement system in tularemia. Our primary focus will be on *F. tularensis* subsp. *tularensis*, although our use of the designation *F. tularensis* reflects an effort to include relevant studies performed with subsp. *holartica* strains. We acknowledge this comes with the risk that future studies may prove some conclusions to be too inclusive.

## Extracellular complement activation and regulation

**Figure 1:**
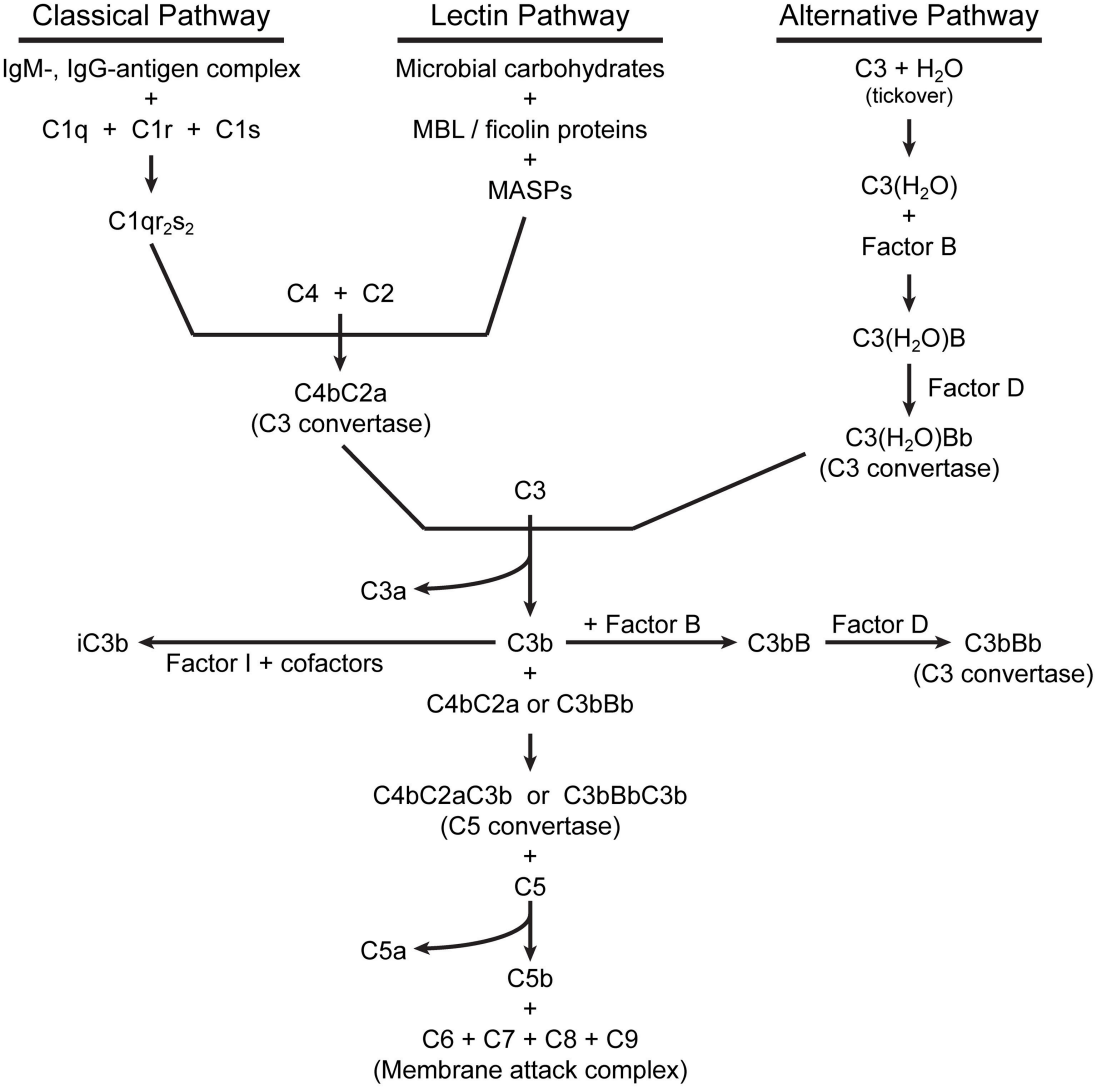
Pathways of extracellular complement activation.

**Figure 2:**
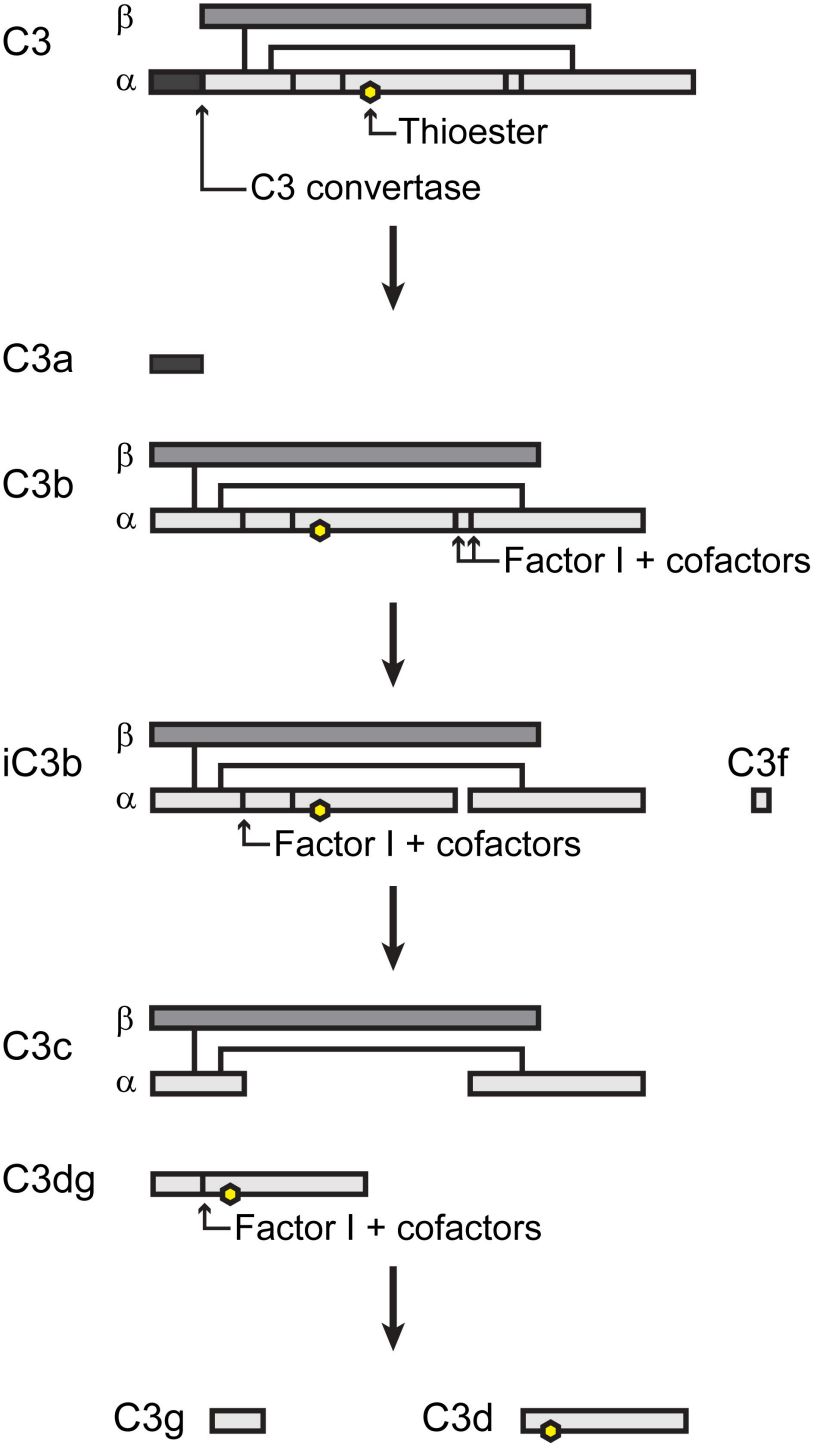
Simple schematic of C3 processing. Adapted from (Nishida et al., 2006).

For detailed descriptions of complement activation, the reader is referred to several excellent reviews (Dunkelberger and Song, 2010; Ricklin et al., 2010; Noris and Remuzzi, 2013). There are three pathways of complement activation (Figure 1), all of which result in the proteolytic cleavage of complement component C3, an abundant serum protein. The classical and lectin pathways both generate a C3 convertase composed of the peptides C4b and C2a. Through the classical pathway, IgM and IgG antibodies, when bound to their respective antigens, bind C1q and initiate the assembly of the C1qr_2_s_2_ complex. This complex cleaves C4 and C2 to produce a C3 convertase, designated C4bC2a. In the lectin pathway, the binding of certain carbohydrate patterns on microorganisms by either serum mannose-binding lectin (MBL) or ficolin proteins recruits and activates mannose-binding lectin-associated serine proteases (MASPs), which cleave C4 and C2 to generate the same C3 convertase. The alternative pathway is constitutively active with a low level “tick-over” of C3 in which an internal thioester bond is spontaneously hydrolyzed yielding C3(H_2_O). Hydrolyzed C3 has a conformation similar to C3b (Chen et al., 2016) and can bind complement factor B (FB). Constitutively active factor D (FD) then cleaves FB, yielding an alternative pathway C3 convertase designated C3(H_2_O)Bb. Like the C4b2a convertase, the alternative pathway convertase can cleave C3 to produce a bioactive short C3a peptide and a lengthier C3b peptide (Figure 2). C3a can serve as an anaphylatoxin. One of the primary functions of C3b is as a potent opsonin, which is attributed to its exposed thioester bond. Unless C3 has been spontaneously hydrolyzed by water, the C3b thioester can react with amine or hydroxyl groups. Formation of amide or ester linkages assures covalent attachment of C3b to nearby target surfaces, which makes them stable ligands for complement receptor-mediated uptake.

The alternative pathway also serves to amplify complement activation initiated by any of the three pathways by utilizing C3b as a focus for the formation of additional C3 convertases. Thus, C3b bound to a surface can form a complex with FB, which when cleaved by FD, becomes the new alternative pathway C3 convertase C3bBb. This amplification process results in increased localized activation of complement in proximity to susceptible microbial surfaces.

The binding of C3b to C3 convertases changes their specificity to C5-cleaving enzymes. Proteolysis of C5 produces the peptides C5a (another anaphylatoxin and potent chemotactic factor) and C5b, which initiates formation of the membrane attack complex. C5b binds C6, which then complexes with C7 and inserts into lipid membranes. The C5b67 complex then interacts with C8, followed by recruitment of multiple copies of C9, which polymerize to form a membrane pore, followed by rapid cell lysis.

Complement activation is tightly regulated by a variety of serum and membrane-bound proteins that control the three complement pathways (reviewed in Zipfel and Skerka, 2009; Noris and Remuzzi, 2013). The primary outcome is to limit host tissue damage that would result from unabated complement activation. Only two of these regulatory components will be mentioned here. Factor I (FI) is a serum protein that, along with certain cofactors, cleaves the alpha chain of C3b yielding inactivated C3b (iC3b) (Figure 2). The formation of iC3b terminates amplification of the complement cascade by preventing formation of additional C3 convertases and also halts C5 cleavage, which diminishes assembly of the membrane attack complex. However, iC3b retains its opsonic activity, albeit with different complement receptor specificity than that of C3b. Factor H (FH) serves as a co-factor for FI and also competes with FB binding to C3b, preventing formation of the alternative pathway C3 convertase.

## Cell surface receptors for C3

In addition to fluid phase complement factors, there are many membrane bound complement receptors that are important for complement regulation and complement-mediated clearance, phagocytosis and cellular signaling. Here we will discuss membrane bound complement receptors that have been shown to have important implications for *F. tularensis* virulence and host defense. For a comprehensive review of complement receptors, the reader is referred to two important reviews (Leslie, 2001; Zipfel and Skerka, 2009). Complement receptor 1 (CR1or CD35) is expressed on leukocytes and erythrocytes and binds C3b (Table 1). In addition to its role in complement-mediated opsonophagocytosis, CR1 serves as a membrane-bound cofactor for FI, leading to conversion of C3b to iC3b. CR3 is a heterodimer of the membrane proteins CD11b and CD18 and is expressed on neutrophils, macrophages, follicular dendritic cells, eosinophils, basophils, NK cells and platelets. CR3 shows high affinity for iC3b, which facilitates phagocytosis of iC3b-bearing particles. While we will focus primarily on the opsonic activities of these receptors, CR3 also plays a role in leukocyte trafficking to sites of infection and regulating cellular responses initiated by certain Toll-like receptors. CR4 is comprised of heterodimers of CD11c and CD18 and is expressed on monocytes and macrophages. CR4 also binds iC3b with high affinity.

**Table 1:** Human complement receptors with known involvement in the uptake of serum-opsonized *F. tularensis*.

**Complement receptor**	Preferred ligand^*^	Cell expression^*^	**Function in *F. tularensis* infection**	**References**
CR1 (CD35)	C3b, C4b	Leukocytes (including neutrophils and macrophages) and erythrocytes	Uptake of serum-opsonized *F. tularensis* by human neutrophils	Schwartz et al., 2012
CR3 (CD11b, CD18)	iC3b	Neutrophils, macrophages, follicular dendritic cells, eosinophils, basophils, NK cells and platelets	Uptake of serum-opsonized *F. tularensis* by human neutrophils, macrophages and dendritic cells Crosstalk with TLR2–inhibition of TLR2-mediated inflammatory signaling	Clemens et al., 2005; Ben Nasr et al., 2006; Schwartz et al., 2012; Dai et al., 2013
CR4 (CD11c, CD18)	iC3b	Monocytes and macrophages	Uptake of serum-opsonized *F. tularensis* by macrophages and dendritic cells	Ben Nasr et al., 2006; Schwartz et al., 2012

* *Receptor ligand specificity and cell expression obtained from Leslie (2001) and Zipfel and Skerka (2009)*.

Apart from the involvement of complement receptors in the uptake of *F. tularensis* by phagocytic cells, little research has investigated the role of other cell surface complement receptors and regulators. For example, tissue macrophages also express the complement receptor CRIg, which binds the beta chain of C3, allowing the receptor to phagocytize both C3b- and iC3b-opsonized particles. CRIg is important for the clearance of pathogens (Helmy et al., 2006; van Lookeren Campagne et al., 2007), but its role during *F. tularensis* infection has not been investigated. Mature B cells express CR2 (or CD21), which binds iC3b, C3dg and C3d peptides derived from FI-mediated cleavage of iC3b. One report suggests that subsets of mouse B cells employ CR2 along with the B cell receptor for uptake of *F. tularensis* (Plzakova et al., 2015). CD46, CD55 and CD59 are expressed by most host cell types, regulate complement activation and protect host tissues from complement-mediated damage by aiding in the inactivation of C3, disrupting the C3 convertases or preventing the formation of the membrane attack complex, respectively. Whether or not these complement regulators play a role in *F. tularensis* virulence or host defense is unknown.

## Intracellular actions of complement

Based on phylogenetic studies and the presence of C3-like proteins in porifera (sponges), Elvington et al. have suggested that complement proteins served first to protect the intracellular space before evolving into a system for defending against pathogens at the cell membrane or in intercellular or intravascular domains of higher organisms (Elvington et al., 2016). Only recently have we begun to appreciate the extent to which complement mediates important intracellular functions (reviewed in Arbore et al., 2017; Liszewski et al., 2017).

Many cell types produce C3 (Lubbers et al., 2017) and maintain intracellular stores of the protein (Liszewski et al., 2013). Elvington et al. recently showed that intracellular C3 derives from a C3(H_2_O) recycling pathway in which hydrolyzed, but not native, C3 is taken up from the extracellular environment (Elvington et al., 2017). After being loaded with C3(H_2_O), Farage B lymphoma cells released ~80% of the C3(H_2_O) back into the culture medium, suggesting that the cells processed the remainder as a source of bioactive C3 peptides (Liszewski et al., 2013; Elvington et al., 2017). Complement receptors CR1, CR2, CR3, and CD46 did not appear to be involved in the uptake of C3(H_2_O) in this recycling pathway (Elvington et al., 2017).

Liszewski and colleagues have extensively documented mechanisms of C3 activation within cells (Liszewski et al., 2013). For example, cathepsin L can cleave C3 to form C3a and C3b within the lysosomes of human T cells. The resulting C3a mediates the tonic intracellular activation of its complement receptor C3aR on the lysosome membrane, leading to baseline mTOR activation necessary for T cell homeostasis. Inhibition of cathepsin L or siRNA inhibition of C3aR expression led to T cell apoptosis. Activation of T cells through their antigen receptors resulted in the transport of vesicles containing C3aR, C3 and cathepsin L to the cell surface. In an autocrine fashion, cleavage of C3 at the cell surface and binding of C3a and C3b to their respective receptors, C3aR and CD46, polarized the T cell to a Th1 phenotype (Liszewski et al., 2013; Kolev et al., 2015).

Intracellular activation of C3 is not limited to T cells. It has also been demonstrated in a variety of primary human cell types including monocytes, neutrophils, and B cells, as well as cultured human fibroblasts, ME-180 epithelial cells and umbilical vein endothelial cells (Liszewski et al., 2013). The proteases responsible for intracellular C3 cleavage vary among cell types. While cathepsin L mediates activation of C3 in T cells and monocytes, it is not responsible for the C3 activation observed in lung epithelial cells (Liszewski et al., 2013). Both cathepsin L and cathepsin B contribute to C3 cleavage within human intestinal epithelial cells (Satyam et al., 2017). Factor H and FI can be taken up by cells and mediate intracellular cleavage of C3(H_2_O) (Elvington et al., 2017). Factor H has also been shown to interact with cathepsin L to increase cleavage of endogenous C3 yielding iC3b (Martin et al., 2016). Clearly, additional studies are needed to form a complete understanding of the significance of intracellular complement activation and its potential relationship with host defense.

Tam and colleagues demonstrated that the presence of C3 peptides in the cytosol may also serve as molecular patterns that initiate danger signaling (Tam et al., 2014). A variety of C3-opsonized microbes, including both RNA and DNA non-enveloped viruses and the Δs*ifA* mutant of *Salmonella*, activated a NF-κB-driven luciferase reporter when present in the cytosol. The reporter was not activated when cytosolic entry was prevented or when pathogens were not opsonized with C3. Latex beads opsonized with a mixture of purified C3, FB and FD also activated NF-κB when transfected into HEK293T cells, suggesting that recognition of microbial patterns was not essential for this response. Signaling initiated by cytosolic C3 was independent of the signaling intermediates MyD88, TRIF, RIG-I, MDA5, Syk, and STING, but appeared to involve the mitochondrial antiviral signaling (MAVS) protein and TNF receptor-associated factor (TRAF). Cytosolic C3 sensing was observed in a variety of non-immune mammalian cell lines indicating that the proposed C3-detection pathway may be active in a number of cell types. However, it remains unknown whether macrophages sense cytosolic C3 in this manner. It should also be noted that these findings have not, as yet, been confirmed by other investigators and that a putative cytosolic C3 sensor has not yet been identified. This laboratory has identified tripartite motif-containing 21 (TRIM21) as a cytosolic sensor for IgG and IgM that also leads to the activation of NF-κB and interferon regulatory factors (James et al., 2007; Mallery et al., 2010; McEwan et al., 2013). The notion that C3 peptides mediate similar intracellular surveillance is quite provocative and certainly worthy of further study.

## Complement activation by *F. tularensis*

In the conventional sense, *F. tularensis* is relatively serum resistant, meaning that it can survive in human serum (HS) without succumbing to the lytic effects of complement (Lofgren et al., 1983; Sorokin et al., 1996). Serum resistance appears to be conferred by the lipopolysaccharide (LPS) and cell wall structure of *F. tularensis*, as evidence by the increased activation of complement (especially via the classical pathway) and susceptibility to serum-mediated lysis of LPS and capsule mutants (Sandstrom et al., 1988; Sorokin et al., 1996; Clay et al., 2008; Lindemann et al., 2011). It is possible that mutations in other *F. tularensis* genes similarly alter the density of iC3b deposition following serum opsonization. Additionally, growth of *F. tularensis* in different culture media, which can alter the expression of high molecular weight surface carbohydrates, can affect the extent of C3 deposition on the bacterial surface (Zarrella et al., 2011). During opsonization of *F. tularensis* in HS, FH can bind to serum-opsonized *F. tularensis* (Ben Nasr and Klimpel, 2008), promoting the conversion of C3b to iC3b. This prevents the assembly of the membrane attack complex on the bacterial surface (Ben Nasr and Klimpel, 2008; Clay et al., 2008). *F. tularensis* may also express a surface CD59-like peptide (Madar et al., 2015), which may further hinder formation of the membrane attack complex by binding C8 or C9.

Several reports indicate that both the classical and alternative pathways are activated by *F. tularensis* (Ben Nasr and Klimpel, 2008; Clay et al., 2008) and include the observation that C1q is required for C3 deposition on the bacterium under certain conditions (Fulop et al., 1993; Clay et al., 2008; Schwartz et al., 2012). Natural IgM antibodies appear to play a role in complement activation by *F. tularensis* via the classical complement pathway (Sandstrom et al., 1988; Schwartz et al., 2012). Schwartz et al. showed that human serum (HS) from donors without a history of tularemia contained IgM antibodies that reacted with *F. tularensis* and mediated C3 deposition during the first 30 min of opsonization. Ben Nasr and Klimpel (2008) reported that, although the classical pathway was activated, they were unable to detect binding of serum IgM to *F. tularensis*. Balagopal et al. (2006) used immunofluorescence and ELISA to detect antibodies bound to *F. novicida* following opsonization of the bacteria with HS. The differences between these reports may reflect the bacterial strains that were studied or the use of different techniques to detect antibody binding. Regardless, there appears to exist sufficient evidence to conclude that the classical pathway can mediate C3 opsonization of *F. tularensis*.

The classical pathway may be particularly important when serum opsonization occurs for periods of <30 min. Longer periods of incubation with serum may allow significant alternative pathway amplification and C3b deposition (Ben Nasr and Klimpel, 2008). We have found that uptake of *F. tularensis* SCHU S4 by human macrophages over 3 h is significantly reduced in C3-depleted HS compared to HS (Brock and Parmely, 2017). However, there is no difference in the level of SCHU S4 uptake during a 3-h incubation in C1q-depleted HS compared to HS (Brock and Parmely, unpublished). Likewise, Ben Nasr and Klimpel (2008) reported that EGTA chelation of Ca^2+^ ions necessary for classical pathway activation in HS delayed the deposition of iC3b on *F. tularensis* if opsonization was limited to 30 min. After 45 min of opsonization, there was no difference between the levels of iC3b deposition that occurred in HS and EGTA-treated HS (Ben Nasr and Klimpel, 2008). Conversely, treatment of HS with EDTA, which chelates both Ca^2+^ and Mg^2+^ and blocks both classical and alternative pathways, prevented any detectable iC3b deposition on SCHU S4 for at least 1 h (Ben Nasr and Klimpel, 2008). Thus, it appears that both the classical and alternative pathways can mediate complement activation during serum opsonization of *F. tularensis*.

## Complement-mediated uptake of *F. tularensis*

For a more comprehensive summary on the role of various cell surface receptors in the uptake of *F. tularensis*, the reader is referred to an excellent review by Moreau and Mann (Moreau and Mann, 2013). Our focus here will be limited to the receptors involved in complement-dependent uptake of *F. tularensis*.

Complement component C3 was first shown to be important for optimal uptake of *F. tularensis* by human monocyte-derived macrophages (MDMs) by replenishing C3-depleted serum with C3 protein (Clemens et al., 2005). This resulted in a C3 concentration-dependent uptake of bacteria. The importance of C3 in the uptake of *F. tularensis* has since been confirmed by several other groups (Dai et al., 2013; Brock and Parmely, 2017). Antibody blocking of CR3 with anti-CD11b and anti-CD18 reduced the uptake of HS-opsonized *F. tularensis* by human MDM (Clemens et al., 2005). Additional studies on blocking of complement receptors with antibodies have demonstrated that CR3 and CR4 are the predominant receptors involved in the uptake of HS-opsonized *F. tularensis* by human macrophages (Schulert and Allen, 2006; Schwartz et al., 2012). However, blocking antibodies often show relatively modest effects in this context. The use of siRNA to inhibit expression of CR3 in human MDM has also demonstrated that CR3 is an important receptor for the uptake of serum-opsonized SCHU S4 (Dai et al., 2013) and is consistent with the observation that C3 deposited on *F. tularensis* during HS opsonization is rapidly converted to iC3b. Inactivated C3b, not C3b, is the primary ligand for CR3 and CR4. CR1 does not appear to play a significant role in the uptake of serum-opsonized *F. tularensis* by human MDM, based on antibody blocking of CR1 (Schwartz et al., 2012).

Another experimental approach for determining important receptor-ligand interactions in C3-mediated uptake of *F. tularensis* has involved heat inactivation of HS to block complement activation or selective depletion of individual complement components, both of which yield greater effects on uptake than receptor blocking with antibodies. Perhaps blocking antibodies lack the affinity required to compete with high affinity natural ligands. Alternatively, incomplete blocking by antibodies to CRs may indicate that other receptors also mediate uptake of serum-opsonized *F. tularensis*. For example, Class A scavenger receptors have been shown to bind iC3b (Goh et al., 2010) and have been implicated in the uptake of serum-opsonized *F. tularensis* (Pierini, 2006; Geier and Celli, 2011). Balagopal et al. (2006) suggested that Fcγ-receptors on human MDM could also contribute to uptake of serum-opsonized *F. novicida*. A role for CRIg in the uptake of serum-opsonized *F. tularensis* has not been investigated. CRIg is a complement receptor expressed on tissue macrophages which binds the beta chain of C3, allowing the receptor to phagocytize both C3b- and iC3b-opsonized particles. The receptor has been shown to be important for the clearance of pathogens (Helmy et al., 2006; van Lookeren Campagne et al., 2007). Thus, although our knowledge of all the receptors that mediate the uptake of serum-opsonized *F. tularensis* may be incomplete, iC3b and CR3 likely play dominate roles in *Francisella* opsonophagocytosis by macrophages.

Complement C3-mediated uptake of *F. tularensis* is not restricted to macrophages. Ben Nasr et al. showed that C3 is also required for increased uptake of *F. tularensis* by human monocyte-derived dendritic cells. Opsonization with C3-depeleted HS resulted in levels of uptake similar to those observed with un-opsonized bacteria (Ben Nasr et al., 2006; Ben Nasr and Klimpel, 2008). Blocking with antibodies to CD11b and CD11c identified CR3 and CR4 as important for enhanced uptake by dendritic cells (Ben Nasr et al., 2006). By contrast, blocking Fc receptors had little effect (Ben Nasr et al., 2006). In similar receptor blocking studies, Schwartz et al. (2012) found that CR1 (CD35) and CR3 (CD11b) mediated uptake of HS-opsonized *F. tularensis* by human neutrophils (Schwartz et al., 2012). These studies illustrate that different cells utilize a range of complement receptors to phagocytize serum-opsonized *F. tularensis*.

## Effects of complement on *F. tularensis* infection of macrophages

Complement C3-opsonization appears to have more effects than simply increasing the number of *F. tularensis* bacteria that are phagocytized. Clemens et al. showed that both non-opsonized and HS-opsonized *F. tularensis* LVS were taken up by a unique process, referred to as “looping phagocytosis,” which involved spacious, asymmetric pseudopod loops (Clemens et al., 2005, 2012). An O-antigen-deficient LVS mutant was also phagocytized via looping in the absence of serum. However, the morphology of uptake of this serum-sensitive O-antigen mutant was altered in the presence of C7-deficient serum, which allowed for opsonization but prevented complement-mediated bacteriolysis (Clemens et al., 2012). C7-deficient serum promoted uptake of the mutant in very tight loops. As serum-sensitive O-antigen mutants support increased C3-deposition (Clay et al., 2008), the authors suggested that an increased interaction between bacterial surface bound C3 peptides and macrophage complement receptors likely led to closer physical interactions at the host-microbe interface (Clemens et al., 2012). An important unanswered question is whether this morphological change leads to different signaling in the host cell.

Geier and Celli demonstrated that CR3 was important in the uptake by mouse bone marrow-derived macrophages (BMM) of HS-opsonized SCHU S4 (Geier and Celli, 2011). Uptake of HS-opsonized SCHU S4 delayed the maturation of the phagosome as measured by the expression of LAMP-1. Baudino et al. have also reported a delay in phagosome maturation associated with the uptake of C3-opsonized apoptotic cells (Baudino et al., 2014). Uptake via CR3 decreased the proportion of SCHU S4 bacteria that escaped phagosomes measured at 30 min PI (Geier and Celli, 2011). However, differences between phagosome escape of HS-opsonized bacteria by wild-type BMM and CD11b-deficient BMM were lost by 45 min PI, suggesting the effect was only temporary.

Geier and Celli also concluded that HS-opsonization restricted the replication of the pathogen measured at 12 h PI. Our own studies with human macrophages indicate that intracellular replication rates of SCHU S4 in human macrophages are not affected by C3-opsonization (Brock and Parmely, 2017). SCHU S4 bacteria taken up in HS did not evidence any impaired ability to replicate to high densities within human primary macrophages. It should be noted that the time required for maximum *F. tularensis* escape from phagosomes appears to be greater in human THP-1 cells and primary macrophages (Clemens et al., 2004) than is observed in murine BMM (Geier and Celli, 2011), and this may explain some of the differences between these studies. Similarly, the percentage of HS-opsonized bacteria that ultimately do escape the phagosome appears to be higher in human macrophages (typically ~80%) (Clemens et al., 2004; Brock and Parmely, 2017) than mouse macrophages (typically ~55%) (Geier and Celli, 2011). Another distinction between these mouse and human studies is the use of human serum as the source of opsonins in both cases. This approach assumes that human C3 interacts with human and mouse complement receptors in a similar manner and that signaling from both species of receptors is also the same.

Dai et al. reported that the binding of C3-opsonized SCHU S4 to CR3 altered the human macrophage response to infection by suppressing inflammatory cytokine production induced by TLR2 (Dai et al., 2013). By comparing infection of MDM with SCHU S4 in C3-depleted and C3-replenished human serum, the investigators found that the presence of C3-opsonization decreased the phosphorylation of MAP kinases ERK and p38 and decreased levels of secreted TNF, IL-6 and IL-1β. Serum opsonization of SCHU S4 also resulted in less NF-κB phosphorylation and nuclear translocation. Using siRNA to inhibit expression of CD11b or TLR2, they demonstrated that TLR2 activated pro-inflammatory responses to *F. tularensis* and that CR3 inhibited TLR2 signaling. CR3 inhibition of TLR2 signaling was mediated through phosphorylation of Lyn kinase.

These studies indicate that the binding and uptake of C3-opsonized *F. tularensis* has a number of effects beyond the promotion of phagocytosis. C3 mediates a different morphology of uptake, significant changes in early host cell signaling pathways, subtle changes in intracellular trafficking and even altered survival of infected macrophages (Brock and Parmely 2017), which will now be discussed in more detail.

## C3 controls macrophage survival during infection with type A *F. tularensis*

While studying infections of human MDM with *F. tularensis* SCHU S4, we observed that large numbers of macrophages in infected cultures died by 24 h PI and that cell death was C3-dependent (Brock and Parmely, 2017). Death was rare among macrophages that had been infected in the presence of heat-inactivated or C3-depleted serum. Single cell analysis by confocal microscopy revealed that a high cytosolic bacterial burden was not required for C3-dependent macrophage death. Many cells that bore only a few bacteria died as long as uptake had been facilitated by the presence of fresh HS. Conversely, half of macrophages that contained more than 100 bacteria did not die by 24 h PI when bacteria were taken up in a C3-dependent fashion. Some MDM in cultures that had been infected with C3-opsonized SCHU S4 lacked any detectable bacteria, and very few of these bystander cells died. Acknowledging that differences in the extent of bacterial uptake existed between the two opsonization conditions, we equalized initial uptake of the pathogen in HS and C3-depleted HS by adjusting the multiplicities of infection (MOI). When initial uptake levels were equivalent, similar bacterial growth occurred under the two conditions, but macrophage death was only seen in the presence of C3. We concluded that high bacterial burden was neither necessary nor sufficient for cell death induction, which was confirmed by infections with the replication-deficient SCHU S4 Δ*purMCD* mutant. Despite its limited intracellular replication, the HS-opsonized Δ*purMCD* mutant strain escaped the phagosome and induced cell death at levels equivalent to those seen in wild type SCHU S4-infected cultures. C3-dependent uptake alone did not explain the induction of macrophage death, as shown by the failure of the phagosome escape-deficient mutant SCHU S4 Δ*fevR* to induce death of MDM, despite C3 opsonization. These findings suggest that two conditions need to be met for macrophage death. First, the cells must contain the pathogen. Second, uptake must occur in a C3-dependent fashion. While we do not yet know all of the details of this process, C3 appears to be emerging as an important factor in the induction of macrophage death that is so commonly seen in tularemia (Parmely et al., 2009).

**Figure 3:**
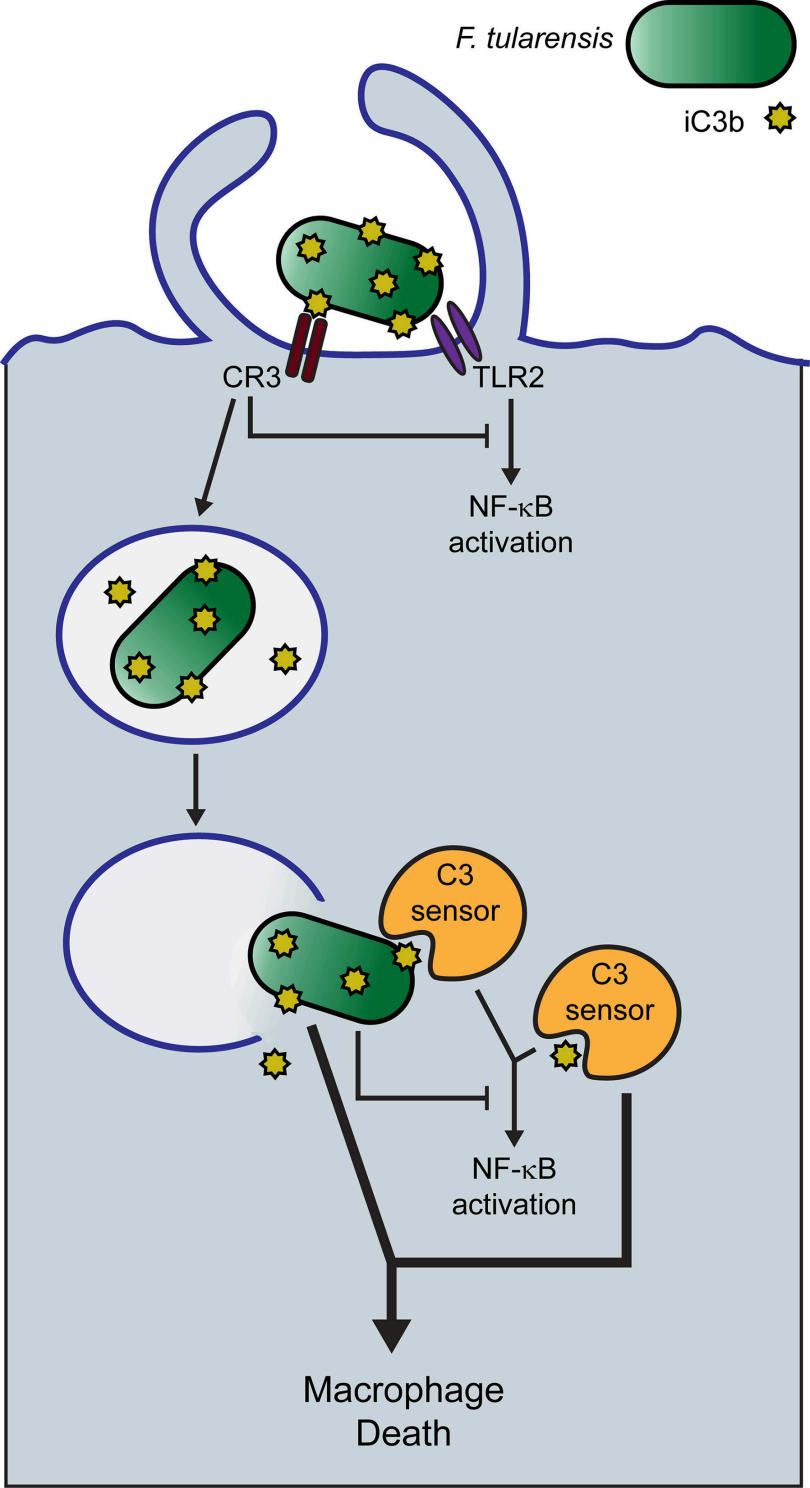
Hypothetical model of serum-opsonized *F. tularensis-*induced human macrophage death. Human serum-opsonized *F. tularensis* delivers C3 peptides into the cytosol of macrophages upon phagosome escape of the pathogen. Cytosolic *F. tularensis* and sensing of cytosolic C3 peptides trigger macrophage death.

The experiments of Tam et al. (2014) reviewed above provide a potential context for understanding how complement promotes macrophage death following infection with type A *F. tularensis*. This group demonstrated that cytosolic C3 peptides, likely in the form of C3b, can activate NF-κB in a number of cell types. If this cellular response was initiated by the sensing of a cytosolic C3 peptide, as postulated by the authors, it would provide a reasonable hypothesis for explaining what we have observed during *F. tularensis* infections of human macrophages. Accordingly, we suggest that C3 peptides, including iC3b, are recognized in the cytosol of macrophages as molecular patterns and that the response to them is directed toward cell death, rather than NF-κB activation, by type A *F. tularensis* (Figure 3). This pathogen has a well-established anti-inflammatory phenotype, which includes its ability to inhibit NF-κB activation (Telepnev et al., 2003, 2005; Bosio et al., 2007; Butchar et al., 2008; Chase et al., 2009; Dotson et al., 2013; Bauler et al., 2014; Ghonime et al., 2015; Putzova et al., 2017). C3-dependent uptake of *F. tularensis* by CR3 further inhibits NF-κB activation and pro-inflammatory gene expression in human macrophages (Dai et al., 2013).

Our prediction that C3 peptides induce macrophage death after SCHU S4 entry into the cytosol rests, in part, on studies with the phagosome escape SCHU S4 Δ*fevR* mutant. Strains deficient in FevR have been used by others to determine the importance of phagosome escape in various aspects of infection of and immunity to *F. tularensis* (Wehrly et al., 2009; Long et al., 2013). However, it should be noted that FevR is a global transcriptional regulator and controls the expression of a number of *F. tularensis* genes.

Our hypothesis would predict that C3 peptides enter the cytosol with *F. tularensis*. Human serum-opsonized *F. tularensis* bears covalently attached C3b and iC3b when it is taken up by cells, although the fate of these peptides during their extended stay in the phagosome is unknown. Phagosome escape by SCHU S4 in human macrophages is not complete until 8 h PI. In this context, it remains unclear whether the pathogen contributes more to macrophage death induction than simply transporting the relevant C3 peptides into the cytosol, but we expect that it does. Tam et al. (2014) were able to elicit a NF-κB response in HEK293T cells by simply transfecting the cells with latex beads opsonized with the purified complement components C3, FB and FD. This would suggest that the NF-κB response does not require a microbial component and that cytosolic C3 peptides may be sufficient for this response. Tam et al. (2014) did not report on the viability of the host cell following the transfection of C3 peptides into the cytosol. Thus, it remains to be determined if cytosolic C3 or cytosolic C3 fragments alone are sufficient to trigger macrophage death. Because both *F. tularensis* itself and CR3 engagement are capable of inhibiting NF-κB activation (Telepnev et al., 2003; Dai et al., 2013; Putzova et al., 2017), we propose that the response to cytosolic C3 in *F. tularensis*-infected macrophages is diverted to a cell death pathway (Figure 3). This would explain the requirement for C3-opsonization and align our findings with the C3 sensing model proposed by Tam and colleagues.

Testing the hypothesis that cell death is initiated by the combined effects of C3 peptides and *F. tularensis* may be best undertaken by the direct delivery of these components into the cytosol of macrophages by methods such as those described by Meyer et al. (2015) or Wu et al. (2015). These experimental approaches would allow one to isolate the effects of the cytosolic microenvironment from those stages of infection preceding phagosome escape and evaluate more precisely the nature of C3, the bacterial components and the host cell recognition process that combines to trigger macrophage death.

What role does CR3, which mediates the uptake of C3-opsonized SCHU S4 by human macrophages, play in signaling cell death? Two observations may be relevant. Dai et al. (2013) showed that human MDM infected with C3-opsonized SCHU S4 produced decreased amounts of IL-1β, a finding we confirmed in our own studies (Brock and Parmely, 2017). Release of IL-1β requires inflammasome-mediated caspase-1 activation, which is not a characteristic of type A *F. tularensis* (Dotson et al., 2013; Ghonime et al., 2015). Thus, it is unlikely that CR3 binding of C3-opsonized SCHU S4 induces caspase-1-mediated pyroptosis in human macrophages as has been reported for mouse macrophages infected with *F. novicida* (Mariathasan et al., 2005; Henry et al., 2007; Peng et al., 2011). A second finding is also relevant. When we first infected human MDM at high MOI with SCHU S4 opsonized with C3-depleted serum and then infected these cells with C3-opsonized SCHU S4 Δ*fevR* mutant bacteria, the infected macrophages remained viable. Secondary infection with C3-opsonized wild type SCHU S4 resulted in macrophage death. Likewise, infection with C3-opsonized SCHU S4 Δ*fevR* alone also did not induce macrophage death, whereas C3-opsonized wild type SCHU S4 alone did. This illustrates that CR3 engagement is not a sufficient death signal, even in macrophages infected with high numbers of intracellular bacteria lacking C3 peptides. A reasonable explanation for these findings is that macrophage death requires both cytosolic *F. tularensis* and cytosolic C3 peptides. While it cannot be ruled out that CR3 signaling (Dai et al., 2013) contributes to macrophage death, there is also no reason to assume that CR3-mediated uptake of C3-opsonized *F. tularensis* is required for cell death induction. CR3 may simply be the most efficient receptor for assuring a high frequency of infected cells.

Although a putative C3 sensor remains to be characterized, two likely ligands—C3b and iC3b—are predicted by available information. First, Tam and his colleagues implied that the ligand was C3b by the few components—C3, FB and FD—that were required for opsonizing latex beads capable of activating NF-κB following their transfection into cells (Tam et al., 2014). Second, when *F. tularensis* SCHU S4 is opsonized with HS, iC3b is the predominant peptide covalently attached to the organism (Ben Nasr and Klimpel, 2008; Clay et al., 2008; Brock and Parmely, 2017). This is consistent with the high levels of uptake of C3-opsonized *F. tularensis* by human macrophages being mediated by CR3 (Clemens et al., 2005; Schwartz et al., 2012; Dai et al., 2013), which shows high affinity for iC3b (Table 1).

Previous studies of mice infected with type A *F. tularensis* revealed a caspase-3-dependent pathway of macrophage death (Parmely et al., 2009; Wickstrum et al., 2009). However, to date, we have been unable to determine the cell death pathway activated by C3-opsonized SCHU S4 in human MDM. The extended period of time between infection with *F. tularensis* and macrophage death has suggested to some that a causal relationship exists between achieving a sufficient intracellular bacterial burden and cell death induction. However, recently published findings (Brock and Parmely, 2017) described above are inconsistent with this interpretation. If the kinetics of cell death reflects an apoptotic process, which can take up to 24 h (Saraste and Pulkki, 2000), then the delay in the appearance of overt signs of cell death (e.g., LDH release) may reflect the variability in apoptosis induction among individual cells, the complex nature of signaling pathways or the lengthy degradative processes necessary for loss of membrane integrity leading to LDH release.

*Francisella tularensis* is likely to encounter the complement system quite early in infection, given the range of cells that produce complement components and the high concentrations of these components in body fluids, especially plasma, alveolar fluids and inflammatory exudates (Schenkein and Genco, 1978; Watford et al., 2000; Holers, 2014; Lubbers et al., 2017). Complement activation by extracellular microbial pathogens has traditionally been viewed as benefiting the host by mediating clearance, leukocyte chemotaxis and altered vascular permeability at sites of infection. However, this view needs to be balanced by recent reports that *F. tularensis* utilizes complement to its own advantage by avoiding many complement effector mechanisms, regulating innate immune cell activation and controlling host cell viability to promote its own survival, intracellular replication and dissemination. Caution is urged in considering therapeutic approaches to infection that might affect complement activation by *F. tularensis*. Clearly, the pathogen has a complicated and largely mysterious relationship with the complement system that deserves additional study to appreciate fully its role in tularemia pathogenesis.

## Ethics statement

This study was carried out in accordance with the recommendations of the human subjects research guidelines of the University of Kansas Medical Center Institutional Review Board with written informed consent from all subjects. All subjects gave written informed consent in accordance with the Declaration of Helsinki. The protocol was approved by the University of Kansas Medical Center Institutional Review Board.

## Author contributions

SB and MP both contributed to the design, conception, and writing of this manuscript.

### Conflict of interest statement

The authors declare that the research was conducted in the absence of any commercial or financial relationships that could be construed as a potential conflict of interest.

## Abbreviations

BMM: bone marrow-derived macrophage
CR: complement receptor
FB: factor B
FD: factor D
FI: factor I
HS: human serum
MDM: monocyte-derived macrophage
MOI: multiplicities of infection
PI: post-infection.
